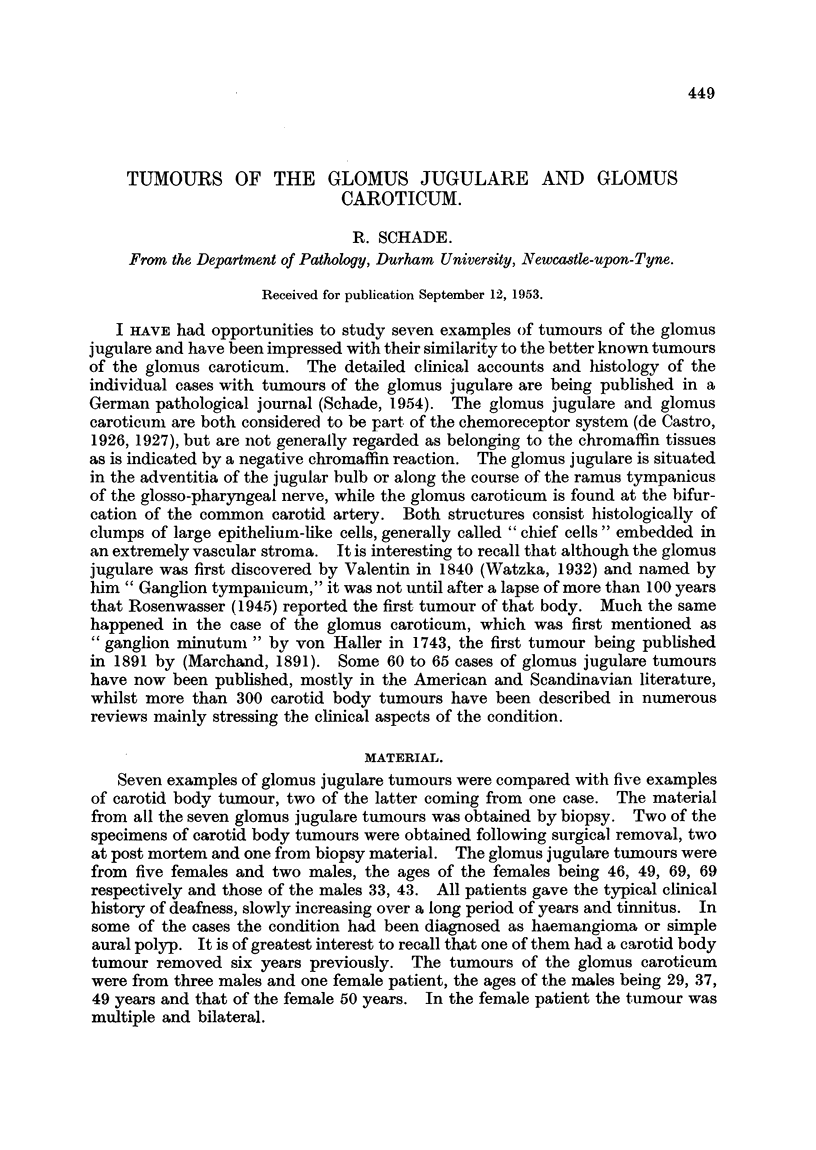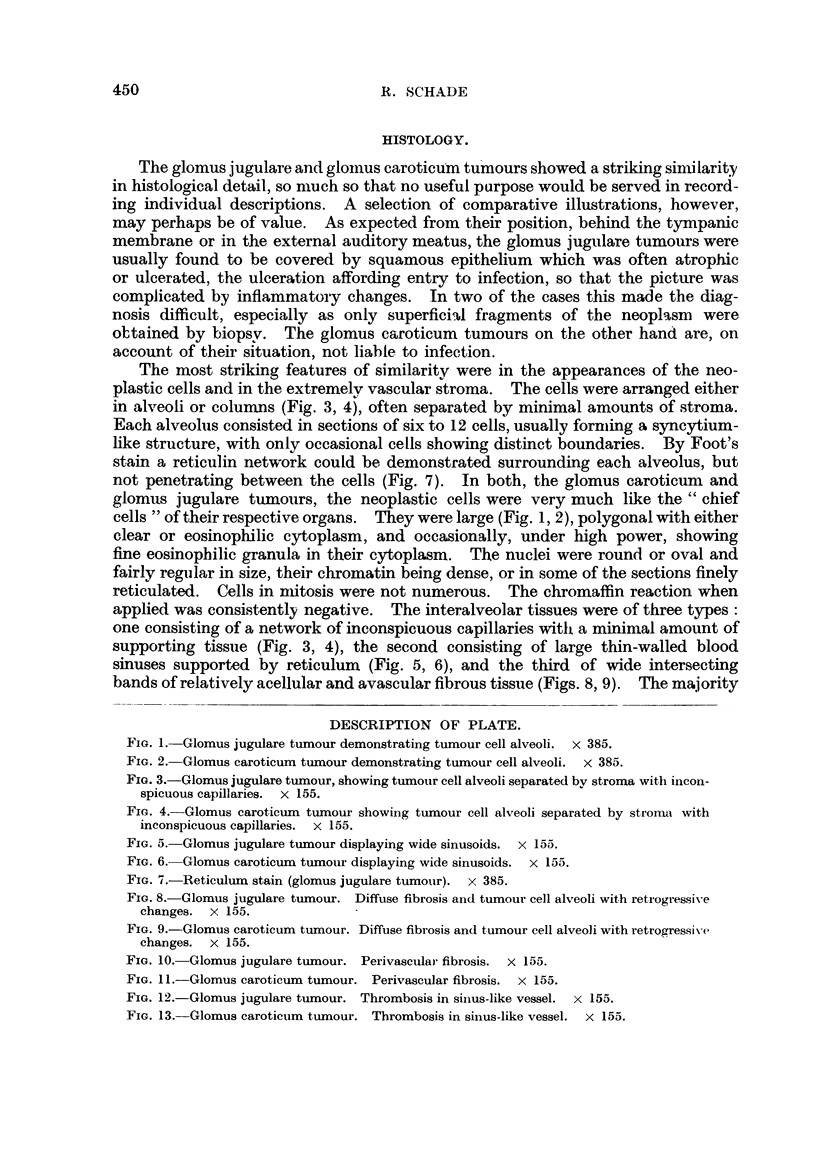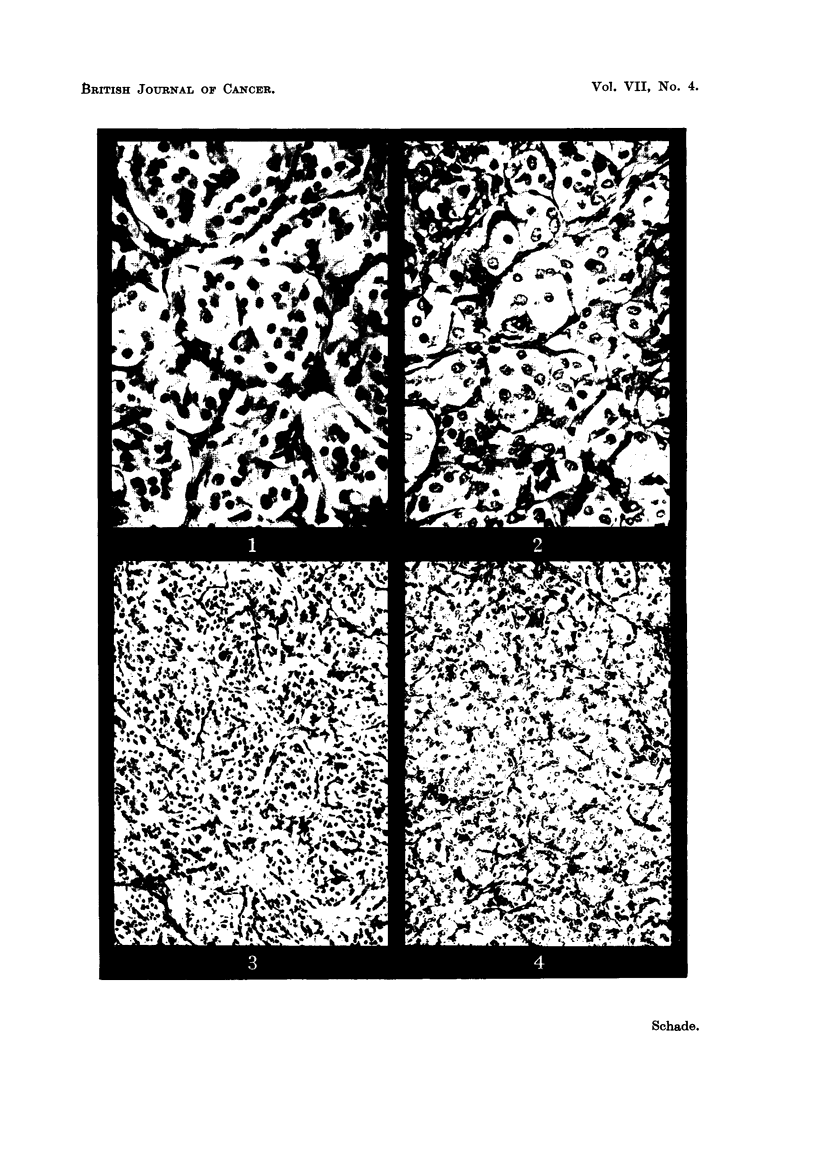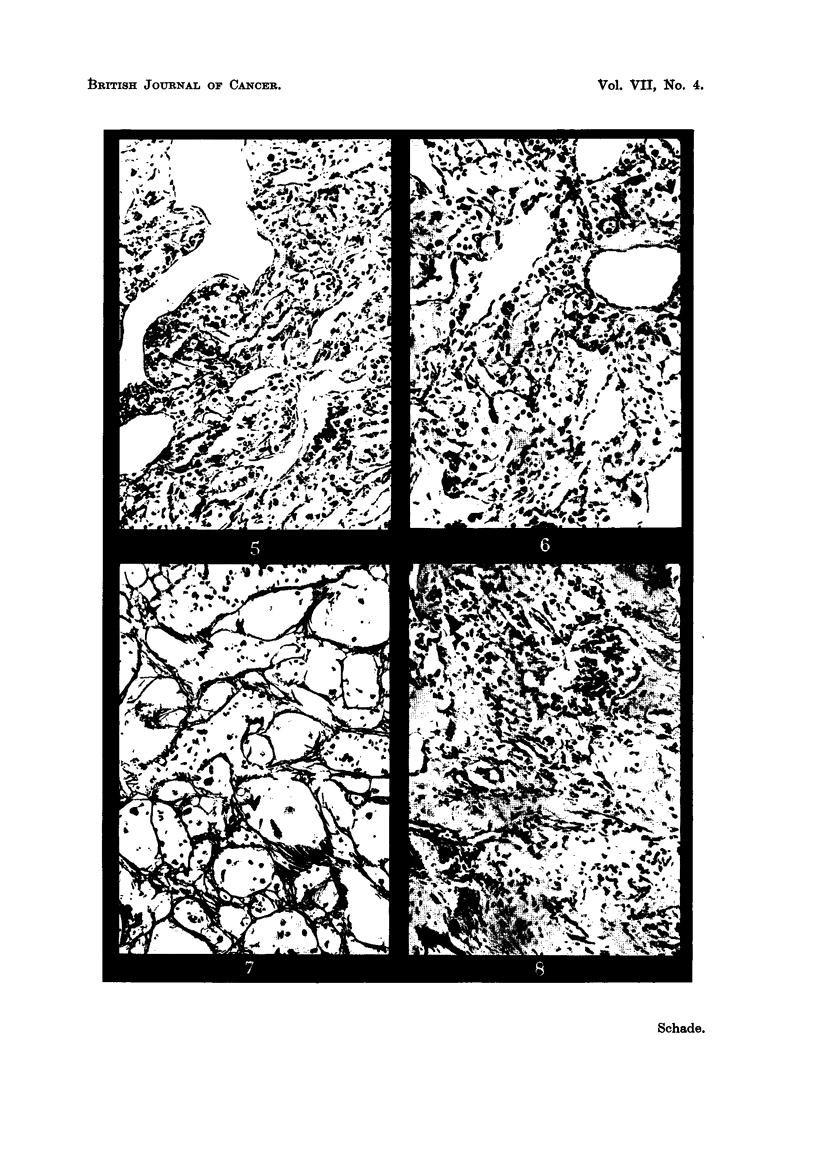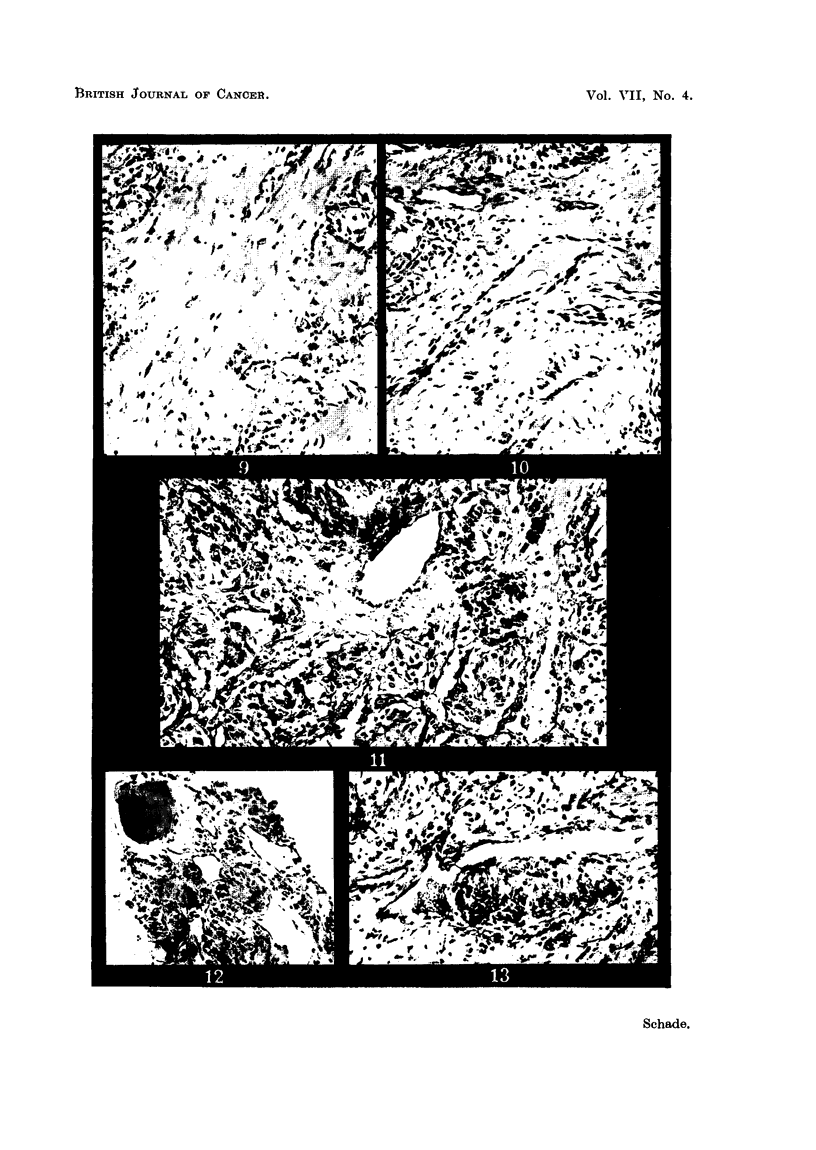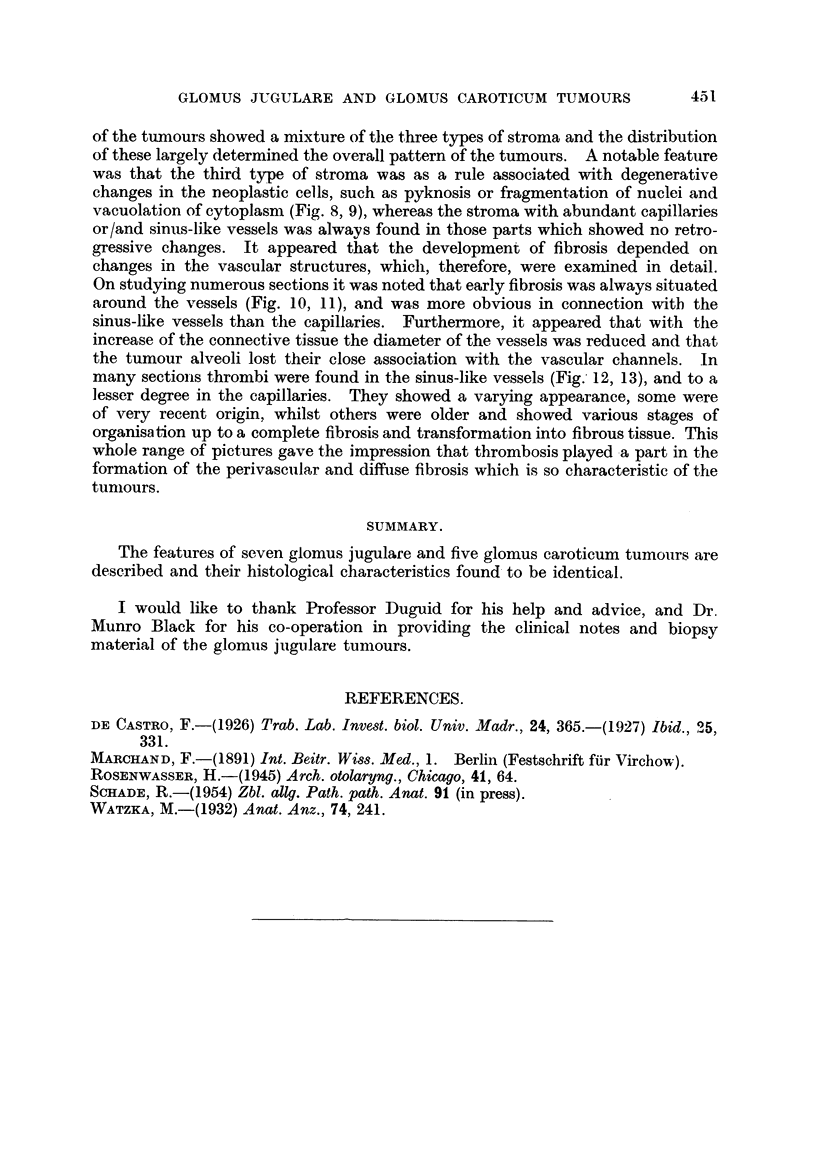# Tumours of the Glomus Jugulare and Glomus Caroticum

**DOI:** 10.1038/bjc.1953.45

**Published:** 1953-12

**Authors:** R. Schade

## Abstract

**Images:**


### TUMOURS OF THE GLOMUS JUGULARE AND GLOMUS CAROTICUM.

R. SCHADE.

*From the Department of Pathology, Durham University, Newcastle-upon-Tyne.*

Received for publication September 12, 1953.

I HAVE had opportunities to study seven examples of tumours of the glomus jugulare and have been impressed with their similarity to the better known tumours of the glomus caroticum. The detailed clinical accounts and histology of the individual cases with tumours of the glomus jugulare are being published in a German pathological journal (Schade, 1954). The glomus jugulare and glomus caroticum are both considered to be part of the chemoreceptor system (de Castro, 1926, 1927), but are not generally regarded as belonging to the chromaffin tissues as is indicated by a negative chromaffin reaction. The glomus jugulare is situated in the adventitia of the jugular bulb or along the course of the ramus tympanicus of the glosso-pharyngeal nerve, while the glomus caroticum is found at the bifurcation of the common carotid artery. Both structures consist histologically of clumps of large epithelium-like cells, generally called "chief cells" embedded in an extremely vascular stroma. It is interesting to recall that although the glomus jugulare was first discovered by Valentin in 1840 (Watzka, 1932) and named by him "Ganglion tympanicum," it was not until after a lapse of more than 100 years that Rosenwasser (1945) reported the first tumour of that body. Much the same happened in the case of the glomus caroticum, which was first mentioned as "ganglion minutum" by von Haller in 1743, the first tumour being published in 1891 by (Marchand, 1891). Some 60 to 65 cases of glomus jugulare tumours have now been published, mostly in the American and Scandinavian literature, whilst more than 300 carotid body tumours have been described in numerous reviews mainly stressing the clinical aspects of the condition.

### MATERIAL.

Seven examples of glomus jugulare tumours were compared with five examples of carotid body tumour, two of the latter coming from one case. The material from all the seven glomus jugulare tumours was obtained by biopsy. Two of the specimens of carotid body tumours were obtained following surgical removal, two at post mortem and one from biopsy material. The glomus jugulare tumours were from five females and two males, the ages of the females being 46, 49, 69, 69 respectively and those of the males 33, 43. All patients gave the typical clinical history of deafness, slowly increasing over a long period of years and tinnitus. In some of the cases the condition had been diagnosed as haemangioma or simple aural polyp. It is of greatest interest to recall that one of them had a carotid body tumour removed six years previously. The tumours of the glomus caroticum were from three males and one female patient, the ages of the males being 29, 37, 49 years and that of the female 50 years. In the female patient the tumour was multiple and bilateral.

### HISTOLOGY.

The glomus jugulare and glomus caroticum tumours showed a striking similarity in histological detail, so much so that no useful purpose would be served in recording individual descriptions. A selection of comparative illustrations, however, may perhaps be of value. As expected from their position, behind the tympanic membrane or in the external auditory meatus, the glomus jugulare tumours were usually found to be covered by squamous epithelium which was often atrophic or ulcerated, the ulceration affording entry to infection, so that the picture was complicated by inflammatory changes. In two of the cases this made the diagnosis difficult, especially as only superficial fragments of the neoplasm were obtained by biopsy. The glomus caroticum tumours on the other hand are, on account of their situation, not liable to infection.

The most striking features of similarity were in the appearances of the neoplastic cells and in the extremely vascular stroma. The cells were arranged either in alveoli or columns (Fig. 3, 4), often separated by minimal amounts of stroma. Each alveolus consisted in sections of six to 12 cells, usually forming a syncytium-like structure, with only occasional cells showing distinct boundaries. By Foot's stain a reticulin network could be demonstrated surrounding each alveolus, but not penetrating between the cells (Fig. 7). In both, the glomus caroticum and glomus jugulare tumours, the neoplastic cells were very much like the " chief cells " of their respective organs. They were large (Fig. 1, 2), polygonal with either clear or eosinophilic cytoplasm, and occasionally, under high power, showing fine eosinophilic granula in their cytoplasm. The nuclei were round or oval and fairly regular in size, their chromatin being dense, or in some of the sections finely reticulated. Cells in mitosis were not numerous. The chromaffin reaction when applied was consistently negative. The interalveolar tissues were of three types : one consisting of a network of inconspicuous capillaries with a minimal amount of supporting tissue (Fig. 3, 4), the second consisting of large thin-walled blood sinuses supported by reticulum (Fig. 5, 6), and the third of wide intersecting bands of relatively acellular and avascular fibrous tissue (Figs. 8, 9). The majority

---

### DESCRIPTION OF PLATE.

FIG. 1.—Glomus jugulare tumour demonstrating tumour cell alveoli. × 385.

FIG. 2.—Glomus caroticum tumour demonstrating tumour cell alveoli. × 385.

FIG. 3.—Glomus jugulare tumour, showing tumour cell alveoli separated by stroma with inconspicuous capillaries. × 155.

FIG. 4.—Glomus caroticum tumour showing tumour cell alveoli separated by stroma with inconspicuous capillaries. × 155.

FIG. 5.—Glomus jugulare tumour displaying wide sinusoids. × 155.

FIG. 6.—Glomus caroticum tumour displaying wide sinusoids. × 155.

FIG. 7.—Reticulum stain (glomus jugulare tumour). × 385.

FIG. 8.—Glomus jugulare tumour. Diffuse fibrosis and tumour cell alveoli with retrogressive changes. × 155.

FIG. 9.—Glomus caroticum tumour. Diffuse fibrosis and tumour cell alveoli with retrogressive changes. × 155.

FIG. 10.—Glomus jugulare tumour. Perivascular fibrosis. × 155.

FIG. 11.—Glomus caroticum tumour. Perivascular fibrosis. × 155.

FIG. 12.—Glomus jugulare tumour. Thrombosis in sinus-like vessel. × 155.

FIG. 13.—Glomus caroticum tumour. Thrombosis in sinus-like vessel. × 155.

Schade.

Schade.

9 10 11 12 13

Schade.

of the tumours showed a mixture of the three types of stroma and the distribution of these largely determined the overall pattern of the tumours. A notable feature was that the third type of stroma was as a rule associated with degenerative changes in the neoplastic cells, such as pyknosis or fragmentation of nuclei and vacuolation of cytoplasm (Fig. 8, 9), whereas the stroma with abundant capillaries or/and sinus-like vessels was always found in those parts which showed no retrogressive changes. It appeared that the development of fibrosis depended on changes in the vascular structures, which, therefore, were examined in detail. On studying numerous sections it was noted that early fibrosis was always situated around the vessels (Fig. 10, 11), and was more obvious in connection with the sinus-like vessels than the capillaries. Furthermore, it appeared that with the increase of the connective tissue the diameter of the vessels was reduced and that the tumour alveoli lost their close association with the vascular channels. In many sections thrombi were found in the sinus-like vessels (Fig. 12, 13), and to a lesser degree in the capillaries. They showed a varying appearance, some were of very recent origin, whilst others were older and showed various stages of organisation up to a complete fibrosis and transformation into fibrous tissue. This whole range of pictures gave the impression that thrombosis played a part in the formation of the perivascular and diffuse fibrosis which is so characteristic of the tumours.

### SUMMARY.

The features of seven glomus jugulare and five glomus caroticum tumours are described and their histological characteristics found to be identical.

I would like to thank Professor Duguid for his help and advice, and Dr. Munro Black for his co-operation in providing the clinical notes and biopsy material of the glomus jugulare tumours.

### REFERENCES.

de Castro, F.—(1926) *Trab. Lab. Invest. biol. Univ. Madr.*, **24**, 365.—(1927) *Ibid.*, **25**, 331.

Marchand, F.—(1891) *Int. Beitr. Wiss. Med.*, 1. Berlin (Festschrift für Virchow).

Rosenwasser, H.—(1945) *Arch. otolaryng., Chicago*, **41**, 64.

Schade, R.—(1954) *Zbl. allg. Path. path. Anat.* **91** (in press).

Watzka, M.—(1932) *Anat. Anz.*, **74**, 241.